# Improving Cancer Classification Accuracy Using Gene Pairs

**DOI:** 10.1371/journal.pone.0014305

**Published:** 2010-12-21

**Authors:** Pankaj Chopra, Jinseung Lee, Jaewoo Kang, Sunwon Lee

**Affiliations:** 1 Department of Human Genetics, School of Medicine, Emory University, Atlanta, Georgia, United States of America; 2 Department of Computer Science and Engineering, College of Information and Communication, Korea University, Seoul, Korea; 3 Department of Biostatistics, College of Medicine, Korea University, Seoul, Korea; Johns Hopkins University, United States of America

## Abstract

Recent studies suggest that the deregulation of pathways, rather than individual genes, may be critical in triggering carcinogenesis. The pathway deregulation is often caused by the simultaneous deregulation of more than one gene in the pathway. This suggests that robust gene pair combinations may exploit the underlying bio-molecular reactions that are relevant to the pathway deregulation and thus they could provide better biomarkers for cancer, as compared to individual genes. In order to validate this hypothesis, in this paper, we used gene pair combinations, called doublets, as input to the cancer classification algorithms, instead of the original expression values, and we showed that the classification accuracy was consistently improved across different datasets and classification algorithms. We validated the proposed approach using nine cancer datasets and five classification algorithms including Prediction Analysis for Microarrays (PAM), C4.5 Decision Trees (DT), Naive Bayesian (NB), Support Vector Machine (SVM), and k-Nearest Neighbor (*k-*NN).

## Introduction

The use of DNA microarrays has resulted in the identification and monitoring of numerous cancer marker genes. These genes have been widely used to differentiate not only cancerous tissue samples from normal healthy ones, but also between different sub-types of cancer [1]–[3]. From a diagnostic point of view, it is important to correctly identify cancerous tissue so that the most appropriate treatment can be given as early as possible.

Numerous classifiers have been proposed and evaluated for their comparative accuracy in correctly identifying cancer tumors [4]–[7]. The most prominent of these classifiers are PAM [8], SVM [9], [10], *k-*NN [11], DT [12], Top Scoring Pair (TSP) [13], and *k-*Top Scoring Pair (*k-*TSP) [6]. The results from these studies indicate that there is no single classifier that has the highest accuracy for all the microarray expression datasets. In this paper, we introduce a novel method that uses gene pairs to improve the overall accuracy of the existing classification methods without altering the underlying algorithms.

Recent research has revealed that biomolecular pathways may be stronger biomarkers for cancer, as compared to the deregulation of individual genes [14]. The deregulation of a different subset of genes, associated with the same pathway, may result in the deregulation of the pathway. Inspecting gene combinations may thus be more effective for cancer classification as compared to independently inspecting individual genes. Motivated by that, the proposed method uses the information derived from the gene pair combinations, instead of the original expression values of the genes. We use the derived information as the input to the existing classification methods. We show that these gene pair combinations, called doublets, consistently improve the classification accuracy of the existing classification algorithms.

The significance of the proposed method is that without changing the underlying classification algorithms we can significantly improve the performance of the algorithms by simply constructing doublets and by using them as input, instead of the raw gene expression values. The doublets can be constructed in various ways. In this paper, we experimented with three different types of doublets: *sumdiff*, *mul* and *sign* doublets. The *sumdiff* doublets are constructed by taking the sum and difference of all pairs of the gene expression vectors such that a doublet is represented as a vector sum or difference of two gene vectors. The *mul* doublets are similarly constructed by taking multiplication, and the *sign* doublets are constructed by taking the signs of the differences of the two gene vectors. Refer to the “Materials and Methods” section for more details.

## Results

*LOOCV* (*Leave One Out Cross Validation*) was conducted to measure the accuracy of doublet-based classification. To test a sample, all the samples, but the tested one, are used to compute the 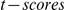 of genes, and the genes are arranged in accordance with the descending absolute values of the scores. The formula used to calculate this score is
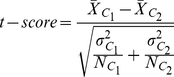
(1)where 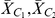 represent the class means; 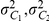 represent the variances; and 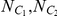 represent the number of samples for the two classes 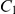 and 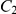, respectively.

We then select the top 0.2%, 0.4%, 0.6%, 0.8%, 1%, 2%, 4%, 10% of the total number of genes in the dataset for making doublets. We further prune the doublets so that no gene appears more than once in the final set of doublets. The algorithm we use to formulate these unique doublets from the original microarray expression dataset is outlined as below.

*Input*: Gene Expression Matrix 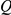 with 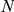 genes and 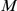 samples, class vector 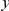 for the 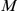 samples and 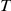 for the number of the genes required for analysis.

*Output*: Unique doublets

 **1.** Compute t-scores for matrix 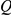 using class vector 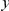.
 **2.** Make an ordered list 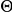 of all the genes 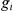, in decreasing value of their absolute t-score.
 **3.** Take the top 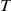 genes from the ordered list 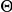, and extract their expression values from 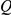. The new expression matrix 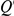 has 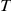 rows and 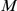 columns.
 **4.** Make doublets from 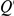 to get a new matrix 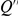, with 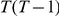 rows and 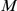 columns.
 **5.** Compute t-scores for matrix 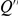 using class vector 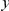.
 **6.** Make an ordered list 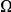 of all the doublets 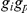 in 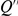, in decreasing value of their absolute t-score.
 **7.** Initialize 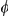 as an empty list.
 **8.** **forall** *doublets* 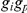 *in* 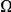 **do** (in decreasing absolute t-score order); If neither of the genes in the doublet 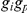 is in 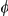, then add doublet 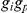 to 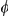

 **9.** Return 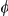

**Figure 1:**
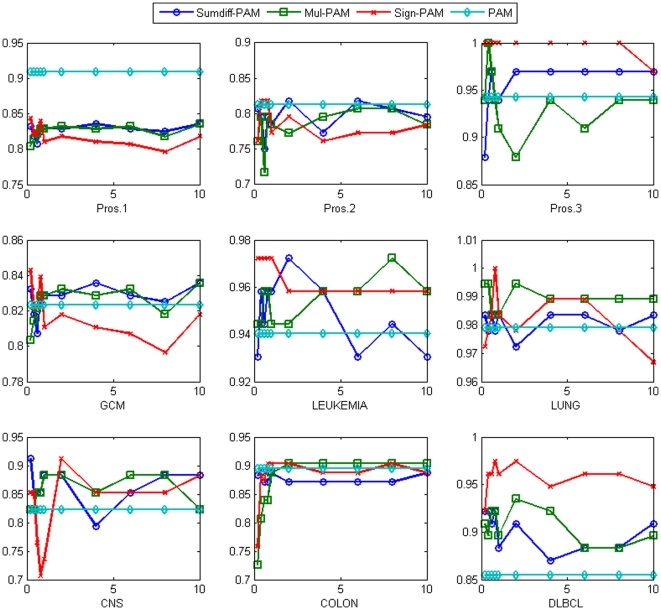
The accuracy of *sumdiff/mul/sign-PAM for the top* 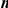
% genes compared with the PAM accuracy for each of the nine datasets.

The accuracy of the original algorithms is measured using all the raw expression values of the genes as input. We shall refer to the accuracy of the original algorithm, for example for PAM, as PAM, and the accuracy obtained using *sumdiff/mul/sign* doublets as input to PAM as *sumdiff/mul/sign-*PAM, respectively. Figure 1 compares the accuracy of the standard PAM classifier to that of *sumdiff/mul/sign-*PAM, obtained by taking the top 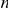
% genes, for the nine datasets listed in Table 1. It can be seen that even taking a small percentage of the top genes and making doublets could improve the performance of PAM. The *sumdiff/mul/sign-*PAM classifier outperforms the standard PAM classifier in many datasets.

**Table 1:** The microarray datasets used for classification.

Dataset	Platform	Total	Total	Reference
		Genes (N)	Samples (M)	
Colon	cDNA	2000	62	Alon [23]
Leukemia	Affy	7129	72	Golub [24]
CNS	Affy	7129	34	Pomeroy [25]
DLBCL	Affy	7129	77	Shipp [26]
Lung	Affy	12533	181	Gordon [27]
Prostate1	Affy	12600	102	Singh [28]
Prostate2	Affy	12625	88	Stuart [29]
Prostate3	Affy	12626	33	Welsh [30]
GCM	Affy	16063	280	Ramaswamy [31]

For the two datasets, CNS and DLBCL, this gain is substantial. For example, with *sign-*PAM using the top 2% genes, the accuracy has increased from 82.4% to 91.2% for the CNS dataset; and for the DLBCL dataset, the accuracy has increased from 85.5% to 97.4%. The average accuracy of the PAM classifier for the nine datasets has increased from 88.7% to 90.6%, 89.3% and 91.7% with *sumdiff*, *mul* and *sign-*PAM with top 2% genes, respectively.

We can make two observations from this result. Only a small number of the top genes are required to achieve improvements and that the improvements are quite consistent across the datasets. In order to show whether or not these observations are still valid for other classification methods, we performed the same experiments using different classification methods including the DT, NB, SVM and *k-*NN classifiers.

**Figure 2:**
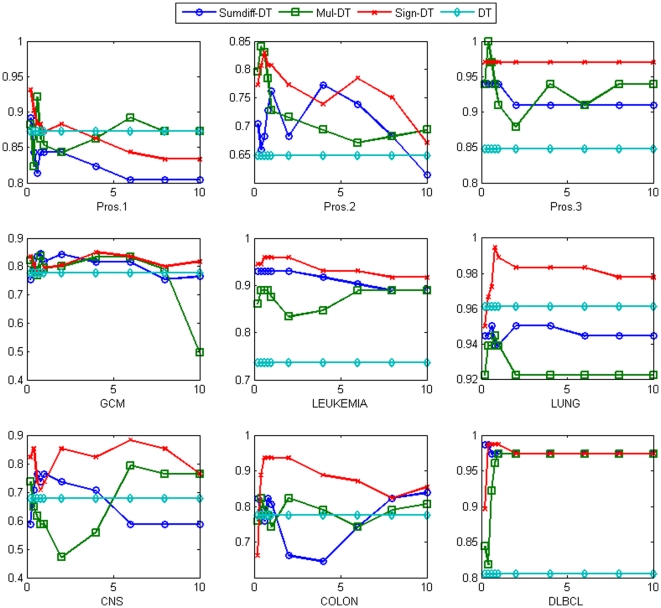
The accuracy of *sumdiff/mul/sign-*DT for the top 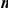
% genes compared with the DT accuracy for each of the nine datasets.

Figure 2 shows the comparison results with DT. The accuracy of DT was consistently improved across the nine datasets. In some cases, the improvements were significant. For example, *sumdiff-*DT improved the accuracy of DT from 64.8% to 77.3% in the Pros.2 dataset using the top 4% genes; from 73.6% to 93.1% in the Leukemia dataset with only the top 0.2% genes; and from 80.5% to 98.7% in the DLBCL dataset with only the top 0.2% genes. Similarly, *mul-*DT improved the accuracy of DT from 64.8% to 84.1% in the Pros.2 dataset using the top 0.4% genes; from 84.9% to 100% in the Pros.3 dataset with the top 0.4% genes; and from 80.5% to 97.4% in the DLBCL dataset with the top 1% genes. Finally, *sign-*DT improved the accuracy of DT from 84.9% to 97.0% in the Pros.3 dataset using the top 0.2% genes; from 73.6% to 95.8% in the Leukemia dataset with the top 0.6% genes; and from 77.4% to 93.6% in the Colon dataset with the top 0.6% genes. On average, over the nine datasets, the accuracy of DT was improved from 78.9% to 85.2%, 84.2% and 89.1% using *sumdiff*, *mul* and *sign* doublets with the top 0.8% genes, respectively.

**Figure 3:**
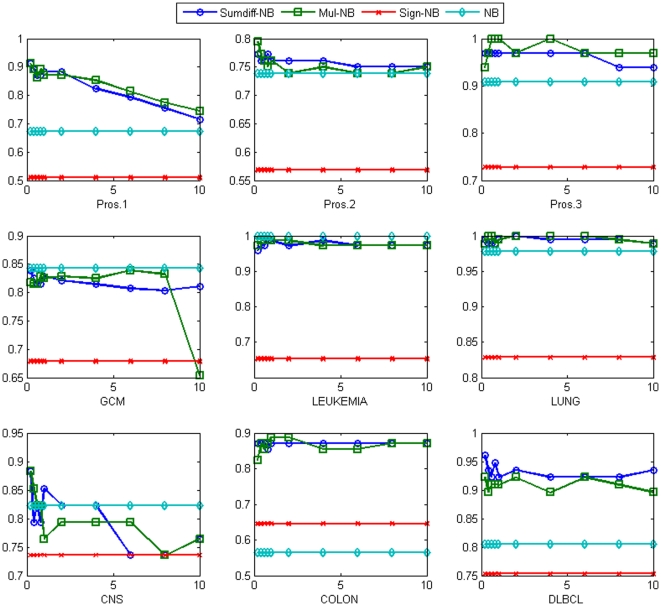
The accuracy of *sumdiff/mul/sign-*NB for the top 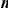
% genes compared with the NB accuracy for each of the nine datasets.

Similarly for NB, the accuracy was significantly improved with *sumdiff* and *mul* doublets. The result is shown in Figure 3. One interesting observation we made is that for NB the *sign* doublets have consistently performed worse than the others independent of the number of the top genes used for doublet generation. This is because the *sign* doublets transform the expression values into binary variables indicating the order of expression level between the genes in the gene pairs and the transformed binary values do not retain enough information to compute the class probability used for classification. Thus, the *sign* doublets are not suitable for the NB classifiers. Nonetheless, the performance gains with *sumdiff* and *mul* doublets were substantial. In the Pros.1 dataset, both *sumdiff/mul-*NB improved the accuracy from 62.8% to 91.2% with the top 0.2% genes; in the Colon dataset, the accuracy was improved from 56.5% to 87.1% and 88.7% with the top 1% genes, respectively. Finally, in the DLBCL dataset, the accuracy was improved from 80.5% to 96.1% and 92.2% with the top 0.2% genes, respectively. On average, the accuracy was improved from 81% to 90.7% and 89.5% with *sumdiff* and *mul* doublets with the top 0.2% genes, respectively.

**Figure 4:**
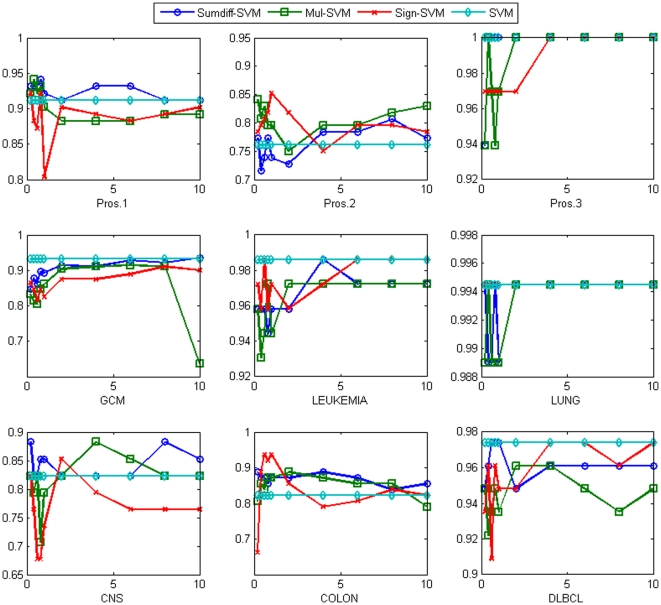
The accuracy of *sumdiff/mul/sign-*SVM for the top 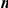
% genes compared with the SVM accuracy for each of the nine datasets.

SVM is known to be one of the most robust classifiers in many domains. Although its performance was compelling by itself, we observed that in some cases our doublet approach improved its performance significantly. The result is shown in Figure 4. In the Colon dataset, the performance gain was most striking. The accuracy was improved from 82.3% to 87.1%, 87.1% and 93.6% with *sumdiff/mul/sign* doublets with the top 1% genes, respectively. In the Pros.2 dataset, the accuracy was improved from 76.1% to 80.7%, 84.1% and 85.2% with the top 8%, 0.2% and 1% genes, respectively. On average, the accuracy was improved from 91.2% to 92%, 91.9%, and 89.4% with *sumdiff/mul/sign* doublets with the top 4% genes, respectively.

**Figure 5:**
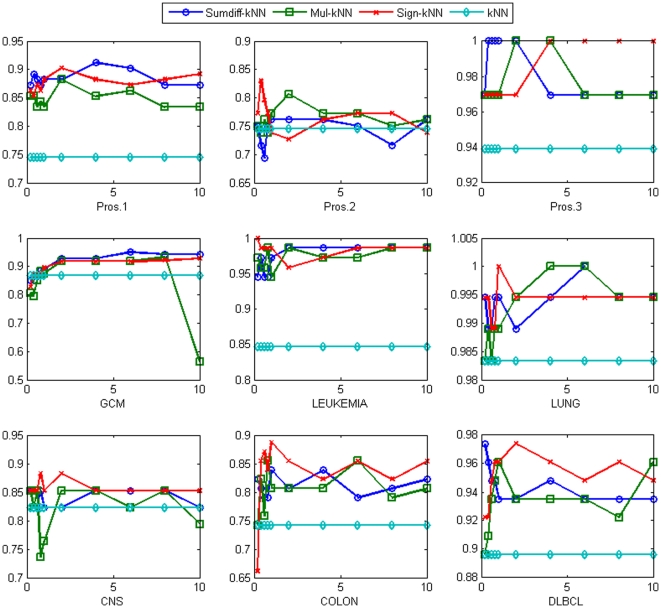
The accuracy of *sumdiff/mul/sign-k-*NN for the top 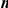
% genes compared with the *k-*NN accuracy for each of the nine datasets.

Lastly, for *k-*NN, the same was observed, as is shown in Figure 5. For *k-*NN, the performance gain was substantial in almost all datasets. For example, in the Leukemia dataset, the accuracy was improved from 84.7% to 98.6%, 98.6%, and 100% with *sumdiff/mul/sign* doublets with the top 2%, 0.8% and 0.2% genes, respectively. On average, the accuracy was improved from 84.3% to 91%, 90.1% and 90.7% with *sumdiff/mul/sign* doublets with the top 4% genes, respectively.

Other than the *sign* doublets in the NB classifier, use of three doublets led to improved performance of the baseline classifiers. The baseline classifiers' average accuracy rates over the nine datasets ranged from 79% to 91% (i.e., DT = 79%, kNN = 84%, NB = 81%, SVM = 91%, and PAM = 89%). On the other hand, their average rates with doublets hovered at a higher range, or from 89% to 92% (i.e., *sign-*DT = 89%, *sumdiff-*kNN = 91%, *sumdiff-*NB = 89%, *sumdiff-*SVM = 92%, and *mul-*PAM = 90%; all the figures with top 4% genes). The baseline classifiers showed a substantial performance difference among them. When it comes to doublets, however, the difference was minimized and the performance was improved. All of the three doublet types almost equally contributed to performance enhancement across various datasets (except the *sign* doublets in the NB). The *sumdiff/mul/sign* doublets with the top 4% genes marked average accuracies over the five classifiers of 88.7% (std. 3.4), 88.5% (std. 3.8), and 85.4% (std. 9.9), respectively. The *sumdiff* doublets demonstrated a slightly better performance than the others did. This result is possibly attributable to the following fact: The *sumdiff* doublets capture both of the upwards and downwards relations (i.e., up-up, down-down, and up-down) and of the order relations of the expression values of each gene pair. On the contrary, the *mul* doublets capture the former alone, and the *sign* doublets capture the latter alone. (See the Materials section for more details.)

## Discussion

A recent study suggested that the pathway level deregulation is more important to carcinogenesis than the deregulation of individual genes [14]. A pathway is typically deregulated by the deregulation of more than one gene that is associated with that pathway. This supports our motivation to use doublets as features for classification, as the doublets could capture potentially more information about the pathway level deregulation than the individual genes. In this study, however, the doublets were pooled from diverse pathways; namely, not limited to those of the gene pairs belonging to the same pathways. By allowing all possible gene combinations, we attempted to capture not only the direct intra-pathway interactions, but also some of the potential indirect inter-pathway associations. We plan to pursue in our future work, the cases where only the intra-pathway doublets are used.

A number of independent studies have attested to the effectiveness of combining gene pairs. Zhou and her colleagues have introduced a technique called *second-order correlation analysis* in which the pair-wise correlations of genes are utilized for the functional classification of genes [15]. Their approach operates, as follows: First computed are all pair-wise correlations of genes within each dataset (1st-order correlations); then, the correlation patterns are analyzed across multiple datasets (2nd-order correlations). Selection is made of the gene pairs that show high correlations in multiple datasets, and the selected form doublets. A doublet is represented as a vector such that its dimension and value, respectively, correspond to a dataset and to the correlation value of the gene pair in the corresponding dataset. The doublets are then clustered using the correlation as similarity metric. The doublets clustered together are considered to share similar functions, because they are turned-on and off collectively across datasets.

We also have developed microarray data integration techniques that exploit inter-gene relations, such as *correlation signature* [16] and *signature cube* [17]. The *correlation signature* projects heterogeneous microarray expression data onto a coherent information space where a gene is represented by the vector of its correlations against a series of landmark genes. If the same set of landmarks is used, heterogeneous microarray datasets, which could not have been directly combined, can be integrated, because the correlation signatures of the genes have compatible dimensions. The *signature cube* generalizes the principles of the correlation signature by providing a heterogeneous microarray data mining framework where data are represented in relative terms (i.e., inter-gene relations). Thus, mining algorithm is coherently applicable all across datasets. Besides the microarray data integration, we also have applied the principle to the clustering problem and have introduced a novel clustering framework, *SignatureClust* [18]. *SignatureClust* clusters microarray data after projecting it into a signature space defined by a set of landmark genes chosen by the user, allowing biologists to get different perspectives of the same underlying data simply by changing the landmark genes.

It also has been proved that the inter-gene information is useful for cancer classification purposes. The *k-*TSP exploits changes in the expression levels of gene pairs in order to improve the classification accuracy [6]. The *k-*TSP classifier uses gene pairs that are similar to our *sign* doublets. The *k-*TSP classifier identifies the gene pairs whose expression orders are consistently reversed across the classes; i.e., if 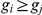 in most of the control samples and 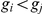 in most of the cancer samples, then the *k-*TSP classifier regards the gene pair 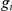 and 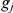 as a good indicator of the classes. The *k-*TSP classifier finds the top-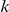 pairs, referred to as TSP (Top Scoring Pairs), and it uses them to determine the classes. The *k-*TSP classifier combines the prediction of each TSP using the unweighted majority voting to determine the final class of a sample. Recently, the *k-*TSP algorithm has also been used to improve the classification accuracy of the SVM classifier [19].

Our method is different from the *k-*TSP classifier in three important aspects. First, 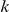-TSP is designed to work with only one type of gene pairing (similar to our *sign* doublets), whereas our method is not limited to specific types of pairing. In this paper, we have defined three doublets, i.e., *sumdiff*, *mul* and *sign*, but various other doublets can also be used with the proposed framework. Second, our method uses existing well established classifiers instead of devising new classification models. This was made possible because our method separates the gene pairing step (i.e., feature extraction step) from the classification model construction. Lastly, the *k-*TSP classifier uses frequency as a metric to assign score to their gene pairs, whereas we use reliable *t-scores*. Table 2 summarizes the accuracy results of the doublets and the baseline classifiers, as well as the accuracy of TSP and *k-*TSP. TSP refers to the case where only the single most influential TSP was used for classification. The TSP and *k-*TSP classifiers reported a robust performance, outperforming most of the baseline classifiers. Still, the two classifiers fall short for the purpose of our study. This study is significant in that it was demonstrated that a simple doublet-based feature extraction method remarkably improves the accuracy of conventional classifiers all the way up to the level of specialized classification algorithms such as TSP and *k-*TSP.

**Table 2:** LOOCV accuracy of the classifiers for the binary class expression datasets.

Method	Leukemia	CNS	DLBCL	Colon	Pros.1	Pros.2	Pros.3	Lung	GCM	Avg.
TSP*	93.80	77.90	98.10	91.10	95.10	67.60	97.00	98.30	75.40	88.26
*k-*TSP*	95.83	97.10	97.40	90.30	91.18	75.00	97.00	98.90	85.40	92.01
DT	73.61	67.65	80.52	77.42	87.25	64.77	84.85	96.13	77.86	78.90
*sumdiff-*DT†	91.67	70.59	97.40	64.52	82.35	77.27	87.88	95.03	81.43	83.13
*mul-*DT†	84.72	55.88	97.40	79.03	86.27	69.32	90.91	92.27	83.21	82.11
*sign-*DT†	93.06	82.35	97.40	88.71	86.27	73.86	96.97	98.34	85.00	89.11
NB	100.00	82.35	80.52	56.45	62.75	73.86	90.91	97.79	84.29	80.99
*sumdiff-*NB†	98.61	82.35	92.21	87.10	82.35	76.14	96.97	99.45	81.43	88.51
*mul-*NB†	97.22	79.41	89.61	85.48	85.29	75.00	100.00	100.00	82.50	88.28
*sign-*NB†	65.28	73.53	75.32	64.52	50.98	56.82	72.73	82.87	67.86	67.77
*k-*NN	84.72	82.35	89.61	74.19	74.51	73.86	93.94	98.34	86.79	84.26
*sumdiff-k-*NN†	98.61	85.29	94.81	83.87	91.18	76.14	96.97	99.45	92.86	91.02
*mul-k-*NN†	97.22	85.29	93.51	80.65	85.29	77.27	100.00	100.00	91.79	90.11
*sign-k-*NN†	97.22	85.29	96.10	82.26	88.24	76.14	100.00	99.45	91.79	90.72
SVM	98.61	82.35	97.40	82.26	91.18	76.14	100.00	99.45	93.21	91.18
*sumdiff-*SVM†	98.61	82.35	96.10	88.71	93.14	78.41	100.00	99.45	91.07	91.98
*mul-*SVM†	97.22	88.24	96.10	87.10	88.24	79.55	100.00	99.45	91.07	91.89
*sign-*SVM†	97.22	79.41	97.40	79.03	89.22	75.00	100.00	99.45	87.5	89.36
PAM	94.03	82.35	85.45	89.52	90.89	81.25	94.24	97.90	82.32	88.66
*sumdiff-*PAM†	95.83	79.41	87.01	87.10	93.14	77.27	96.97	98.34	83.57	88.74
*mul-*PAM†	95.83	85.29	92.21	90.32	92.16	79.55	93.94	98.90	82.86	90.12
*sign-*PAM†	95.83	85.29	94.81	88.71	90.20	76.14	100.00	98.9	81.07	90.11

*Results obtained in [6]

† Results from taking the top 4% of genes for making unique doublets.

The top 15 doublets and their associated KEGG pathways for the CNS dataset are shown in Table 3. One possible explanation on why the doublet accuracy is higher than those of the baseline classifiers could be that the pathways associated with each element of the doublet are somehow interlocked with each other, and therefore form a more robust biomarker compared to each of the pathways taken individually. However, a more robust investigation is required before any hypothesis can be validated. In our future work, we intend to conduct a systematic analysis of these top doublets, their associated pathways and their possible links to cancer.

**Table 3:** KEGG pathways related to the top 15 doublets for the CNS dataset.

Doublet No.	Probe 1	Gene 1	KEGG 1	Probe 2	Gene 2	KEGG 2
1	U40317_s_at	PTPRS	Unknown	U27459_at	ORC2L	Cell cycle
2	J00212_f_at	IFNA21	Cytokine-cytokine receptor interaction	U33920_at	SEMA3F	Axon guidance
			Regulation of autophagy			
			Antigen processing and presentation			
			Toll-like receptor signaling pathway			
			Jak-STAT signaling pathway			
			Natural killer cell mediated cytotoicity			
			Autoimmune thyroid disease			
3	D50924_at	DHX34	Unknown	X04707_at	THRB	Neuroactive ligand-receptor interaction
4	U31215_s_at	GRM1	Calcium signaling pathway	M64929_at	PPP2R2A	Tight junction
			Neuroactive ligand-receptor interaction			
			Gap junction			
			Long-term potentiation			
			Long-term depression			
5	U52828_s_at	CTNND2	Unknown	U33267_at	GLRB	Neuroactive ligand-receptor interaction
6	D50582_at	KCNJ11	Type II diabetes mellitus	Y10204_at	Unknown	Unknown
7	U83600_at	TNFRSF25	Cytokine-cytokine receptor interaction	HG2260-HT2349_s_at	Unknown	Unknown
8	S77835_s_at	IL2	Cytokine-cytokine receptor interaction	M60858_rna1_at	NCL	Pathogenic Escherichia coli infection - EHEC
			Jak-STAT signaling pathway			Pathogenic Escherichia coli infection - EPEC
			T cell receptor signaling pathway			
			Type I diabetes mellitus			
			Autoimmune thyroid disease			
			Allograft rejection			
			Graft-versus-host disease			
9	L32179_at	AADAC	Alkaloid biosynthesis II	M14660_at	IFIT2	Unknown
10	D50310_at	CCNI	Unknown	L78833_cds2_at	RND2	Unknown
11	HG2417-HT2513_at	Unknown	Unknown	U35451_at	CBX1	Unknown
12	U03090_at	PLA2G5	Glycerophospholipid metabolism	M16594_at	GSTA2	Glutathione metabolism
			Ether lipid metabolism			Metabolism of enobiotics by cytochrome P450
			Arachidonic acid metabolism			Drug metabolism - cytochrome P450
			Linoleic acid metabolism			
			alpha-Linolenic acid metabolism			
			MAPK signaling pathway			
			VEGF signaling pathway			
			Fc epsilon RI signaling pathway			
			Long-term depression			
			GnRH signaling pathway			
13	U68536_at	ZNF24	Unknown	X64728_at	CHML	Unknown
14	L43964_at	PSEN2	Notch signaling pathway	X98206_at	Unknown	Unknown
			Alzheimers disease			
15	D13118_at	ATP5G1	Oxidative phosphorylation	U78556_at	MTMR11	Unknown
			Alzheimers disease			
			Parkinsons disease			

We have shown that combining the expression data from gene pairs increases the accuracy of classifiers. We also have shown that increasing the number of genes for making doublets does not necessarily result in a commensurate increase in accuracy. This is significant because we can get a very high accuracy even though we use a very small subset of the total number of genes. Thus, the computational complexity of computing doublets, which can potentially be quadratic to the total number of genes in the dataset, is not critical since only a very small subset of the genes is used.

The genes comprising the top doublets also provide easily interpretable results, as compared to other methods like SVM. Although SVM may provide a higher accuracy than others, it is essentially a black box and no insight can be gained regarding biomarker genes. Doublets, on the other hand, are easily interpretable. Doublets identify which genes and which gene pairs can serve as biomarkers for tumor classification.

In the future, we plan to analyze these doublets across datasets and cancer types to select more robust cancer biomarker gene pairs. Especially, we will investigate how the individual doublets map to real genes' relations, such as suppression or stimulation, and how the relations function with regard to the carcinogenesis. It is further intended to exam the effectiveness of doublets in classifying multi-class cancer datasets.

### Conclusion

The contribution of this paper is twofold. First, it has introduced doublets, a novel method to combine expression data from gene pairs. Gene pairs are more robust biomarkers as compared to individual genes, perhaps reflecting the fact that genes are interacting to perform a molecular function and the deregulation of the genes in the interaction, rather than independent genes, may be responsible for deregulating the critical pathways. Second, we have combined doublets with conventional classifiers to produce classifiers whose accuracy is greater than that of the original ones. We validated the framework using five well-known classifiers including PAM, DT, NB, SVM, and kNN. We showed that doublets can be easily incorporated into the existing classifiers without having to change the underlying algorithms, and that using doublets can consistently improve the classification accuracy of the original algorithms across different datasets.

## Materials and Methods

### Gene Doublets

Let there be *N* genes 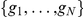 in a tissue sample, and let there be *M* such tissue samples 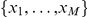. The cancer dataset could then be represented as matrix 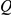 of dimension 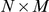. Then, 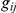 would denote the expression value of the *i*-th gene, 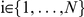 in the *j*-th sample, 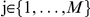. The gene vector 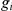  =  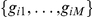 would denote the expression value of the *i*-th gene across the *M* tissue samples, and the column vector 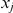  =  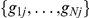 would represent the *j*-th tissue sample across the *N* genes. The class labels for the tissue samples are represented by vector 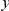  =  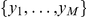, where 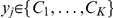, the set of all class labels. For our binary classification problem, 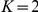, where 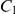 denotes cancerous and 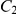 denotes normal tissue samples.

For each pair of genes 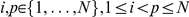 in a dataset, we define a positive doublet vector and a negative doublet vector as
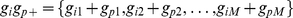
(2)

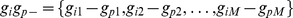
(3)


Thus, for our dataset with 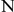 genes, we have 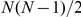 positive doublets and 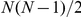 negative doublets, and our original microarray dataset of dimension 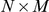 is transformed into an 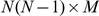 matrix. Each row in this new matrix represents a doublet (positive or negative). We denote this matrix as 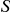, with dimension 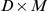, where 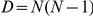; thus, the defined doublets are known as *sumdiff* doublets. In another variation of making doublets, we define the *mul* doublets as:
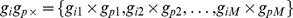
(4)and *sign* doublets as:
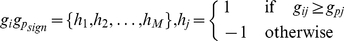
(5)


The *sumdiff* doublets capture up-up, down-down (i.e. positive doublets) and up-down (i.e. negative doublets) relations of the expression values of gene pairs. Furthermore, the negative doublets capture the order of expression values between the genes in the gene pair. Please be noted that the datasets were processed to have a minimum value of 10 and a maximum of 16,000. Thereafter, the values were converted through 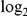. Then, all the samples were standardized to zero mean and unit variance. The *mul* doublets not only capture the up-up, down-down, and up-down relations of gene pairs, but also amplify the relations through multiplication. However, the *mul* doublets do not capture the expression orders between genes. On the other hand, the *sign* doublets capture the inter-gene expression orders alone.

### Microarray Data and Classification Methods

The microarray data is taken from several studies, as is shown in Table 1. These are the same datasets that were used in [6] for comparing TSP and *k-*TSP with various classifiers. The microarrays consist of the expression data for the tissues associated with colon, blood, lung, breast, prostate, and cancer of the central nervous system. The number of samples and the number of genes in each study are also shown in Table 1. For the baseline classifiers, we used the implementations available in Bioconductor (for PAM) [20] and Weka (for DT, NB, SVM and kNN) [21].

### Classification Accuracy

We use the *LOOCV* (*Leave One Out Cross Validation*) method to estimate the classifier accuracy. For each sample 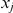 in the dataset, we use the rest of the 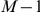 samples in the dataset to predict the class of the 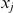 sample. The classification accuracy of each dataset is the ratio of the number of the correctly classified samples (True Positives+True Negatives) to the total number of samples 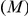 in that dataset.
